# Assessing the Impact of Laparoscopic Cholecystectomy on Satisfaction, Quality of Life, and Cost-Effectiveness in Saudi Patients With Gallstone Disease: A Comprehensive Cross-Sectional Analysis

**DOI:** 10.7759/cureus.45288

**Published:** 2023-09-15

**Authors:** Abdulsalam Aleid, Zainah A Alhebshi, Emad S Alhazmi, Renad M Alshehri, Alhassan Almonawar, Zahrah N Ahmed, Abbas Al Mutair, Mohammed Y Alessa, Loai S Albinsaad

**Affiliations:** 1 Department of Neurosurgery, King Faisal University, Al-Ahsa, SAU; 2 College of Medicine and Surgery, Batterjee Medical College, Jeddah, SAU; 3 College of Medicine and Surgery, Taibah University, Al-Madinah Al-Munawwarah, SAU; 4 Department of Surgery, King Khalid University, Abha, SAU; 5 College of Medicine, King Khalid University, Abha, SAU; 6 College of Medicine and Surgery, Fakeeh College of Medical Sciences, Jeddah, SAU; 7 Research Center, Almoosa Specialist Hospital, Al-Ahsa, SAU; 8 Department of Surgery, College of Medicine, King Faisal University, Al-Ahsa, SAU

**Keywords:** saudi patients, cost-effectiveness, quality of life, satisfaction, gallstone disease, laparoscopic cholecystectomy

## Abstract

Introduction: Cholelithiasis, or gallstone disease, is a prevalent medical condition with substantial global implications. Laparoscopic cholecystectomy (LC) has become the dominant surgical solution for treating various biliary conditions in affluent countries due to its numerous benefits, such as shorter hospital stays and reduced costs. An assessment of postoperative recovery, patient satisfaction, and quality of life (QoL) is crucial to judge the success of any medical procedure regarding long-term patient well-being. Given the scarcity of research on the satisfaction, QoL, and cost-effectiveness of LC among Saudi patients, this study seeks to fill this gap.

Methods: To achieve the study's objectives, a cross-sectional research study was conducted from January to December 2023, focusing on Saudi patients who had received LC for gallstone disease. We utilized an extensive questionnaire to determine patient satisfaction, QoL, and the perceived value of LC, which combined closed and open-ended questions to provide a holistic understanding. Additionally, an in-depth literature review was performed to compare our findings with existing research.

Results: Our survey received answers from 886 Saudi LC patients. Generally, participants showed satisfaction with LC, though complications were reported in a significant number of cases. However, a minority of participants were dissatisfied. Most respondents indicated a moderate enhancement in their QoL postsurgery. Notably, demographic factors like gender, age, and employment status had profound effects on satisfaction and QoL, with male participants more likely to report higher satisfaction and QoL than females.

Conclusion: Our data firmly support the ongoing use of LC as the preferred surgical technique for treating biliary diseases in Saudi Arabia. They emphasize the benefits of personalizing care based on patient demographics to improve the overall experience. Proper communication, thorough preoperative planning, and attentive postoperative care are essential for achieving the best outcomes. Despite these findings, more research is needed, focusing on different patient demographics and comparing LC with other treatment methods to enhance our understanding of gallstone disease management in the Saudi context.

## Introduction

Gallstone disease, or cholelithiasis, covers a variety of conditions, such as asymptomatic cholelithiasis, biliary colic, and complicated gallstones like empyema gallbladder, gangrene, and even peritonitis [1]. The inflammation of the gallbladder, cholecystitis, stands as the primary reason for right upper quadrant (RUQ) pain, leading it to be one of the foremost causes for emergency surgical admissions [2]. With the evolution of medical procedures, laparoscopic cholecystectomy (LC), a minimally invasive surgery for removing a diseased gallbladder, has overtaken the traditional open technique since the early 1990s [3]. This procedure is now the first choice for a range of conditions, from symptomatic cholelithiasis and chronic cholecystitis to more serious conditions like gallstone pancreatitis [4]. In high-income countries, LC's dominance is evident due to the numerous benefits it offers [5], such as reduced hospital stays, quicker recovery, and cost savings [6]. With an impressively low mortality rate of 0.22-0.4%, its safety is largely affirmed [7].

A vital area of focus in medical research nowadays is the health-related quality of life (HRQoL). While its definition might remain broad, encompassing physical, emotional, and social dimensions, its relevance has surged, especially when assessing medical interventions [8]. The outcomes as reported by patients, including pain and quality of life (QoL), become pivotal for a surgeon when considering a procedure [9]. Notably, the gastrointestinal quality of life index (GIQLI) is frequently employed to measure QoL for biliary tract diseases, indicating an improvement in gastrointestinal symptoms post-LC [10].

In the context of Saudi patients, we posit two hypotheses. First, we anticipate that those undergoing LC will exhibit greater satisfaction compared to patients subjected to other treatments. Additionally, LC is expected to bring about a marked enhancement in the QoL of these patients vis-à-vis alternative treatment modalities. To validate these hypotheses, we will employ a slew of statistical techniques, from correlation and regression analysis to the chi-square test.

Guided by the above, the core objectives of this study center around gauging patient satisfaction post-LC for gallstone disease, understanding its impact on Saudi patients' QoL, and weighing its cost-effectiveness relative to other treatment options.

## Materials and methods

Study location

The research was conducted in Saudi Arabia, specifically in areas known for high gallstone disease incidence and frequent LC interventions.

Study design and duration

A cross-sectional approach was adopted from the first of January to the 15th of August 2023. Data were primarily gathered via questionnaires distributed to Saudi patients who had experienced LC due to gallstone disease.

Participants

The subjects of interest were Saudi patients who had received LC across various hospitals in Saudi Arabia.

Sample size and selection

A power analysis determined the necessary sample size, factoring in a 95% confidence level and a 5% margin of error. Through convenience sampling, patients were selected based on their availability and consent to participate.

Inclusion criteria

Saudi patients, aged 18 and above, who had previously undergone LC for gallstone disease were included.

Exclusion criteria

Those who underwent open cholecystectomy, declined participation, or could not provide informed consent were omitted.

Data gathering instruments

A standardized questionnaire was the principal tool, aimed at assessing patient satisfaction, QoL postprocedure, and perceived cost-effectiveness of LC. This questionnaire incorporated both open-ended and closed questions for an exhaustive grasp of the patients' views.

Variables

Independent Variables

Variables such as age, gender, educational attainment, and employment status were deemed independent.

Dependent Variables

Key outcomes included patient satisfaction, postoperative QoL, and perceived cost-effectiveness.

Preliminary study

To evaluate the reliability and validity of the questionnaire, a pilot study with 30 participants was conducted. For validity, feedback from participants prompted minor adjustments to enhance clarity and organization of some questions.

For reliability, we assessed the internal consistency of the 'Satisfaction' and 'Quality of Life' sections using Cronbach's alpha. The results, as illustrated in Table 1, demonstrate good internal consistency, particularly for the 'Quality of Life' section.

**Table 1:** Internal consistency of questionnaire sections

Section	Number of items	Cronbach's alpha
Satisfaction	10	0.782
Quality of Life	11	0.881

The outcomes suggest that our questionnaire exhibits both satisfactory validity and reliability.

Ethical stipulations

Ethical clearance was secured from pertinent local ethics committees. All participants were briefed on the study's objectives and offered informed consent. Ensuring anonymous responses upheld confidentiality. No conflicts of interest were detected in this study.

Constraints

This study faced potential limitations, such as the possibility of biased self-reported data. Due to the specific sampling technique and regional emphasis, results may not represent the entire Saudi patient population.

Data analysis

IBM SPSS Statistics for Windows, Version 28 (Released 2021; IBM Corp., Armonk, New York, United States) facilitated the analysis. Descriptive statistics, like frequencies and percentages, portrayed the demographics of the 886 respondents. Age, gender, educational background, employment status, and regional data were analyzed. Quantitative responses on satisfaction and postsurgery QoL were articulated using means and standard deviations. Inferential statistics, especially logistic regression models, probed the connection between demographic attributes and central outcomes. The questionnaire's reliability was confirmed by computing the Cronbach's alpha for the Satisfaction and Quality of Life portions, thus guaranteeing internal consistency.

## Results

Among 886 participants in Table 2, the majority of the 886 participants were in the 35-44 age range (56.5%), with smaller proportions in the 25-34 (24.9%) and 45-54 (7.9%) groups. The gender distribution was nearly equal, with 52.5% female and 47.5% male participants. Educational attainment showed that a significant portion held bachelor’s degrees (73.4%), followed by high school or lower education (12.4%). The majority of participants were employed full-time (23.2%) or unemployed (35.6%), while other employment statuses constituted smaller percentages. Regarding geographic distribution, the South Province accounted for the largest share (67.8%), followed by the Urban population (83.6%). 

**Table 2:** Demographic characteristics This table provides an overview of the key demographic characteristics of the study participants, including age, gender, education level, employment status, and geographic distribution.

		Count	%
Age	18-24	90	10.2%
	25-34	220	24.9%
	35-44	500	56.5%
	45-54	70	7.9%
	More than 65	5	0.6%
Gender	Female	465	52.5%
	Male	420	47.5%
Education level: Employment Status	Bachelor ‘s degree	650	73.4%
	Diploma	45	5.1%
	Doctorate	10	1.1%
	high school or less	110	12.4%
	Master’s degree	70	7.9%
City of residence	Employed full-time	205	23.2%
	Employed part-time	235	26.6%
	Other	20	2.3%
	Retired	5	0.6%
	Student	105	11.9%
	Unemployed	315	35.6%
Geographic location	Eastern province	75	8.5%
	Middle province	85	9.6%
	Northern province	25	2.8%
	Other	10	1.1%
	South Province	600	67.8%
	Western province	90	10.2%
	Rural	50	5.6%
	Suburban	95	10.7%
	Urban	740	83.6%

General information regarding LC

Table 3 shows that participants' overall satisfaction with the procedure was quite favorable, with 61.6% reporting satisfaction and an additional 25.4% being very satisfied. However, a minority expressed dissatisfaction (2.3%). Complications or adverse effects were experienced by 39.5% of participants. Notably, 64.4% did not experience recurrence of gallstone-related symptoms after the procedure.

**Table 3:** General information regarding laparoscopic cholecystectomy This table presents participants' responses to general questions about their satisfaction with the laparoscopic cholecystectomy procedure, complications experienced, improvement in quality of life, postoperative pain, limitations in daily activities, and perceived financial burden.

		Count	%
How would you rate your overall satisfaction with the laparoscopic cholecystectomy procedure?	Dissatisfied	20	2.3%
	Neutral	95	10.7%
	Satisfied	545	61.6%
	Very satisfied	225	25.4%
Did you experience any complications or adverse effects following the laparoscopic cholecystectomy procedure?	No	535	60.5%
	Yes	350	39.5%
How would you rate the improvement in your quality of life since undergoing laparoscopic cholecystectomy	Decline	5	0.6%
	Moderate improvement	550	62.1%
	No Change	10	1.1%
	Significant improvement	245	27.7%
	Slight improvement	75	8.5%
Compared to your expectations, how would you rate the postoperative pain after laparoscopic cholecystectomy?	As expected,	185	20.9%
	Less Than expected	285	32.2%
	More Than expected	170	19.2%
	Much Less Than Expected	200	22.6%
	Much More Than Expected	45	5.1%
Did you experience any limitations or difficulties in performing daily activities after laparoscopic cholecystectomy?	No	560	63.3%
	Yes	325	36.7%
How satisfied are you with the postoperative follow-up care provided by healthcare professionals?	Dissatisfied	30	3.4%
	Neutral	165	18.6%
	Satisfied	510	57.6%
	Very dissatisfied	5	0.6%
	Very satisfied	175	19.8%
Have you experienced any recurrence of gallstone-related symptoms after laparoscopic cholecystectomy?	No	570	64.4%
	Yes	315	35.6%
How would you rate the financial burden associated with the laparoscopic cholecystectomy procedure	Affordable	400	45.2%
	Expensive	160	18.1%
	Neutral	215	24.3%
	Very affordable	95	10.7%
	Very Expensive	15	1.7%
Did you have any pre-existing conditions that affected your recovery after laparoscopic cholecystectomy?	No	570	64.4%
	Yes	315	35.6%
How well were you informed about the potential risks and benefits of laparoscopic cholecystectomy before the procedure	Moderately Informed	130	14.7%
	Not Informed at All	30	3.4%
	Poorly Informed	45	5.1%
	Very Well Informed	265	29.9%
	Well Informed	415	46.9%

Table 4 displays participants rated their overall satisfaction with the communication between them and the surgical team before the procedure, with a mean score of 4.12 (SD = 1.2) indicating a very high satisfaction of the procedure. The clarity of instructions provided for postoperative care was rated as 3.88 (SD = 0.983). Satisfaction with nursing care during the hospital stay was reported as 3.57 (SD = 1.049). Importantly, the majority of participants would recommend LC to others based on their personal experience (mean = 3.79, SD = 0.954).

**Table 4:** General information regarding laparoscopic cholecystectomy This table presents participants' responses to general questions about their satisfaction with the laparoscopic cholecystectomy procedure, complications experienced, improvement in quality of life, postoperative pain, limitations in daily activities, and perceived financial burden.

		Count	%
How would you rate your overall satisfaction with the laparoscopic cholecystectomy procedure?	Dissatisfied	20	2.3%
	Neutral	95	10.7%
	Satisfied	545	61.6%
	Very satisfied	225	25.4%
Did you experience any complications or adverse effects following the laparoscopic cholecystectomy procedure?	No	535	60.5%
	Yes	350	39.5%
How would you rate the improvement in your quality of life since undergoing laparoscopic cholecystectomy	Decline	5	0.6%
	Moderate improvement	550	62.1%
	No Change	10	1.1%
	Significant improvement	245	27.7%
	Slight improvement	75	8.5%
Compared to your expectations, how would you rate the postoperative pain after laparoscopic cholecystectomy?	As expected,	185	20.9%
	Less Than expected	285	32.2%
	More Than expected	170	19.2%
	Much Less Than Expected	200	22.6%
	Much More Than Expected	45	5.1%
Did you experience any limitations or difficulties in performing daily activities after laparoscopic cholecystectomy?	No	560	63.3%
	Yes	325	36.7%
How satisfied are you with the postoperative follow-up care provided by healthcare professionals?	Dissatisfied	30	3.4%
	Neutral	165	18.6%
	Satisfied	510	57.6%
	Very dissatisfied	5	0.6%
	Very satisfied	175	19.8%
Have you experienced any recurrence of gallstone-related symptoms after laparoscopic cholecystectomy?	No	570	64.4%
	Yes	315	35.6%
How would you rate the financial burden associated with the laparoscopic cholecystectomy procedure	Affordable	400	45.2%
	Expensive	160	18.1%
	Neutral	215	24.3%
	Very affordable	95	10.7%
	Very Expensive	15	1.7%
Did you have any pre-existing conditions that affected your recovery after laparoscopic cholecystectomy?	No	570	64.4%
	Yes	315	35.6%
How well were you informed about the potential risks and benefits of laparoscopic cholecystectomy before the procedure	Moderately Informed	130	14.7%
	Not Informed at All	30	3.4%
	Poorly Informed	45	5.1%
	Very Well Informed	265	29.9%
	Well Informed	415	46.9%

Categories of satisfaction

Table 5 categorizes the participants' satisfaction levels. The majority of participants (63.6%) reported being very satisfied with the LC procedure, while 29.8% indicated being satisfied. A smaller proportion (9.5%) expressed not being satisfied.

**Table 5:** Satisfaction scale This table outlines participants' ratings on various aspects related to their experience with laparoscopic cholecystectomy.

	N	Minimum	Maximum	Mean	SD
How would you rate the communication between you and the surgical team before the procedure?	886	1	5	4.12	1.2
Were you given adequate information regarding the preoperative preparations for laparoscopic cholecystectomy?	886	1	5	3.96	1.129
How would you rate the responsiveness of healthcare professionals to your concerns and questions before the procedure?	886	1	5	3.75	1.061
How would you rate the clarity of instructions provided for postoperative care?	886	1	5	3.88	.983
Did you feel supported by the healthcare team during the recovery period after laparoscopic cholecystectomy?	886	1	5	3.70	.944
How satisfied were you with the information provided about potential complications and their management after the procedure?	886	1	5	3.5	.988
How would you rate the overall professionalism and competence of the healthcare team involved in your laparoscopic cholecystectomy?	886	1	5	3.79	.954
Did the healthcare team address your pain management needs effectively after laparoscopic cholecystectomy?	886	1	5	3.72	1.134
How satisfied were you with the nursing care provided during your hospital stay for laparoscopic cholecystectomy?	886	1	5	3.57	1.049
Would you recommend laparoscopic cholecystectomy to others based on your personal experience?	886	1	5	3.79	.954

Factors influencing satisfaction

Tables 6, 7 demonstrate that gender (p = 0.01), age (p = 0.008), education level (p < 0.001), employment status (p = 0.001), and city of residence (p = 0.031) were found to significantly influence overall satisfaction levels. Notably, male participants had higher odds of being satisfied compared to females (OR = 1.24, 95% CI = 1.20 - 1.91). Participants with a master’s degree had significantly lower odds of being satisfied compared to those with a high school or lower education (OR = 1.30, 95% CI = 0.42 - 1.88). Employment status also played a significant role, with employed full-time participants having lower odds of satisfaction compared to other employment categories (OR = 0.30, 95% CI = 0.42 - 0.88).

**Table 6:** Categories of satisfaction This table categorizes participants' overall satisfaction levels into "Very satisfied," "Satisfied," and "Not satisfied" based on their responses to the satisfaction scale questions.

	Number	%
Very satisfied	563	63.6%
satisfied	264	29.8%
Not satisfied	84	9.5%

**Table 7:** Factors influencing satisfaction This table presents the association between demographic factors (gender, age, education level, employment status, city of residence) and overall satisfaction with the laparoscopic cholecystectomy procedure. The chi-square test was used to explore the statistical significance of potential comparisons according to the level of satisfaction, a p-value less than 0.05 is significant.

Satisfaction		Very satisfied	Satisfied	Not satisfied	p-value
Gender	Male	65%	30%	5%	0.01
	Female	2.3%	89.3%	8.4%	
Age	18-24	60%	30%	10 %	0.008
	25-34	87.9%	0.8%	11.3%	
	35-44	6.7%	82.7%	10.7%	
	45-64	0	100.0%	0	
	65 or more				
Educational level	Bachelor ‘s degree	3.6%	90.4%	6.0%	0.02
	Diploma	1.5%	88.3%	10.2%	
	Doctorate	1.9%	92.3%	5.8%	
	high school or less	92.7%	2..7%	7.3%	
	Master’s degree	58.1%	33.9%	8%	
Employment status	Employed full-time	70%	22.7%	7.3%	0.001
	Employed part-time	80%	11.1%	8.9%	
	Other	60%	32.5%	7.5%	
	Retired	50%	39.4%	10.6	
	Student	59.6%	30%	10.4	
	Unemployed	70%	25.8	4.2%	
City of residence:	Eastern province	80%	16.2%	3.8%	0.031
	Middle province	70%	27.7%	2.3%	
	Northern province	80%	17.4%	2.6%	
	Other	51.0%	40%	9.0%	
	South Province	68.3%	30%	1.7%	
	Western province	92.7%	2..7%	7.3%	
Geographic location	Rural	58.1%	33.9%	8%	
	Suburban	70%	22.7%	7.3%	
	Urban				

Self-reported QoL after the procedure

Table 8 shows the overall physical well-being after the procedure received a mean score of 3.92 (SD = 0.974). The majority reported moderate improvement (62.1%) and significant improvement (27.7%) in their quality of life. Satisfaction with the ability to engage in physical activities was generally high (mean = 4.00, SD = 1.000).

**Table 8:** Logistic regression model of factors influencing the overall satisfaction level This table shows the odds ratios and confidence intervals for the logistic regression model exploring the impact of demographic factors on overall satisfaction with the laparoscopic cholecystectomy procedure.

Variable	Odds Ratio (OR)	95% Confidence Interval (CI)	P-value
Gender (Reference: Female)	1.24	1.20 - 1.91	<0.001
Education level			<0.001
- High school or less	1 (Reference)		
- Diploma	0.83	0.68 - 1.33	
-Doctorate	0.92	0.70 – 1.22	
- Bachelor’s degree	0.71	0.61 - 1.05	
- Master’s degree	1.30	0.42 - 1.88	
Employment Status			<0.001
- Employed full-time	1 (Reference)		
- Employed part-time	1.17	0.74 - 1.85	
- Other	2.21	1.44 - 3.40	
- Retired	1.65	1.26 - 2.17	
- Student	0.71	0.61 - 1.05	
- Unemployed	0.30	0.42 - 0.88	
Geographic Location			0.326
- Urban	1 (Reference)		
- Rural	1.32	0.85 - 2.04	
- Suburban	0.98	0.74 - 1.30	

Factors influencing QoL

Tables 9-11 show that gender (p = 0.04), age (p <0.001), education level (p < 0.001), employment status (p = 0.001), and city of residence (p <0.001) were found to significantly influence overall satisfaction levels. Gender (p < 0.001), education level (p < 0.001), employment status (p < 0.001), and city of residence (p < 0.001) were found to significantly influence self-reported QoL. Male participants had higher odds of reporting a higher quality of life compared to females (OR = 1.54, 95% CI = 1.20 - 1.98). Participants with a master’s degree had significantly higher odds of reporting a higher quality of life (OR = 0.60, 95% CI = 0.42 - 0.85).

**Table 9:** Self-reported quality of life after the procedure This table displays participants' self-reported ratings on various aspects of their quality of life following the laparoscopic cholecystectomy.

	N	Minimum	Maximum	Mean	SD
How would you rate your overall physical well-being after laparoscopic cholecystectomy?	886	1	5	3.92	.974
Did you experience any improvements in your digestion and appetite after laparoscopic cholecystectomy?	886	1	5	3.83	1.007
How satisfied are you with your ability to engage in physical activities after laparoscopic cholecystectomy?	886	1	5	4.00	.000
How would you rate your mental and emotional well-being after laparoscopic cholecystectomy?	886	1	5	3.97	1.007
Have you noticed any changes in your sleep patterns or quality of sleep since undergoing laparoscopic cholecystectomy?	886	1	5	4.00	1.000
How satisfied are you with your overall energy levels and vitality after laparoscopic cholecystectomy?	886	1	5	3.92	.974
Did laparoscopic cholecystectomy improve your ability to perform daily activities and tasks?	886	1	5	3.88	.983
How would you rate your social interactions and relationships after laparoscopic cholecystectomy?	886	1	5	3.70	.944
How would you rate your social interactions and relationships after laparoscopic cholecystectomy?	886	1	5	3.5	.988
Have you experienced any negative effects on your body image or self-esteem following laparoscopic cholecystectomy?	886	1	5	3.79	.954
Overall, how satisfied are you with your quality of life after undergoing laparoscopic cholecystectomy?	886	1	5	3.88	.983

**Table 10:** Association between demographic characteristics and self-reported quality of life. This table examines the relationship between demographic factors (gender, age, education level, employment status, city of residence) and self-reported quality of life after the laparoscopic cholecystectomy procedure.

				P-value
		Mean	SD	
Gender	Female	3.71	.68	0.04
	Male	3.88	1.12	
Age	18-24	4.05	.35	<0.001
	25-34	3.08	1.21	
	35-44	3.51	.79	
	45-54	3.94	.66	
	55-64	3.90	.85	
	Above 65	3.82	1.11	
Education Level	Bachelor ‘s degree	4.01	.69	<0.001
	Diploma	4.43	.60	
	Doctorate degree	3.72	.80	
	high school or less	2.65	2.33	
	Master’s degree	4.73	.29	
Employment Status :	Employed full-time	3.08	1.21	<0.001
	Employed part-time	3.05	.56	
	Other	3.07	.59	
	Retired	4.02	.21	
	Student	3.66	.91	
	Unemployed	3.90	.72	
Residency	Eastern province	3.35	1.24	<0.001
	Middle province	3.08	1.21	
	Northern province	4.01	.69	
	Other	4.43	.60	
	South Province	3.72	.80	
	Western province	2.65	2.33	
Geographic Location	Rural	3.73	.29	<0.001
	Suburban	3.73	.29	
	Urban	4.08	1.21	

**Table 11:** Factors influencing the self-reported quality of life after surgery This table presents the odds ratios and confidence intervals for the logistic regression model investigating the impact of demographic factors on self-reported quality of life after the laparoscopic cholecystectomy procedure.

Variable	Odds Ratio (OR)	95% Confidence Interval (CI)	P-value
Gender (Reference: Female)	1.54	1.20 - 1.98	<0.001
Education level			<0.001
- High school or less	1 (Reference)		
- Diploma	0.92	0.68 - 1.25	
- Bachelor’s degree	0.78	0.61 - 1.01	
- Master’s degree	0.60	0.42 - 0.85	
Employment Status			<0.001
- Employed full-time	1 (Reference)		
- Employed part-time	1.17	0.74 - 1.85	
- Other	2.21	1.44 - 3.40	
- Retired	1.65	1.26 - 2.17	
- Student	1.32	1.06 - 1.64	
- Unemployed	1.14	0.88 - 1.47	
Geographic Location			0.326
- Urban	1 (Reference)		
- Rural	1.32	0.85 - 2.04	
- Suburban	0.98	0.74 - 1.30	
Alcohol Consumption			0.04
- Rarely or never	1 (Reference)		
- Occasionally	1.68	1.10 - 2.57	
- Daily	1.12	0.61 - 2.05	

## Discussion

The purpose of this cross-sectional study was to look into the QoL and satisfaction levels of Saudi patients who had LC for gallstone disease. The questionnaire, primarily divided into four sections, demographic information, general questions, satisfaction level, and quality of life, can be found in Table 12 present in the appendices. Our research findings substantially confirm the hypotheses that LC improves patients' QoL and satisfaction levels significantly more than alternative treatments. These findings are consistent with both international and regional literature, indicating that LC improves patients' QoL [6,8]. Gallstone disease is one of the most common medical issues, with different degrees of severity and complications. LC, a minimally invasive surgical operation that removes the damaged gallbladder, is the standard treatment for symptomatic cholelithiasis and chronic cholecystitis, offering advantages such as a reduced hospital length of stay and lower perioperative morbidity and mortality. However, little evidence has been reported on postoperative changes in QoL following LC. To test these hypotheses, appropriate statistical methods, including correlation analysis, and Chi-square test, were utilized.

Interestingly, the study discovered no significant relationship between patients' age and their evaluation of postsurgery QoL. This finding contrasts with previous research that showed older age was related to worse results from surgery [7].

It is worth noting that the Middle East results indicate a female majority in the patient distribution undergoing LC. However, our study found no significant gender differences in postsurgery satisfaction. Although a previous study has suggested that women may have more severe QoL and gallstone symptoms and recover more slowly than men [8], our data suggest that gender has no effect on reported QoL after LC.

Our review of Saudi Arabian data revealed a need for more comprehensive and localized research on LC's cost-effectiveness. While early laparoscopic cholecystectomy appears to be more cost-effective than delayed laparoscopic cholecystectomy in high-income countries, there is a gap in the literature regarding other economies, including Saudi Arabia [9]. Such research could provide valuable insights for health policy and planning and management.

The findings of the study highlight the importance of patient-reported outcomes in evaluating surgical treatments. While standard measurements of outcomes like mortality and complication rates provide important information regarding the safety and efficacy of surgery, patient-reported outcomes can shed information on the procedure's effect on patients' everyday lives and general well-being.

Furthermore, our findings indicate that the minimally invasive procedure of cholecystectomy, particularly LC, should stay the primary line of surgical care in Saudi Arabia for simple and complex biliary diseases. This is because it is associated with a shorter hospital stay, decreased perioperative morbidity and mortality, a faster return to work, and lower total hospital costs [10].

In conclusion, while this study provides useful insights into the QoL after LC among Saudi patients, more research is needed to comprehensively investigate the experiences of different patient demographics and compare LC with alternative treatment options. To further optimize patient care, future research could look into the elements that affect the perceived QoL after surgery, such as physical, emotional, and social factors.

## Conclusions

In conclusion, this cross-sectional study underscores the positive impact of LC on the QoL and satisfaction levels among Saudi patients with gallstone disease. Notably, while our findings concur with global and regional literature that LC enhances QoL, they also illuminate that factors such as age and gender may not significantly influence post-operative QoL perceptions in the Saudi context. Despite LC's evident benefits, such as reduced hospital stays and lower costs, there remains an evident gap in localized research concerning its cost-effectiveness in economies like Saudi Arabia. The results accentuate the value of patient-reported outcomes, going beyond conventional metrics like mortality rates to understand a procedure's holistic influence on a patient's life. Looking forward, as LC continues to be the preferred surgical approach for biliary diseases in Saudi Arabia, further investigations are essential to delve deeper into various patient experiences and holistically evaluate LC against other treatments. The ultimate goal should be to continuously refine and enhance patient care by understanding the multifaceted factors affecting postsurgical QoL.

## Appendices

Table 12 reflects the questionnaire used in this study.

**Table 12:** Postprocedure patient experience and satisfaction survey: laparoscopic cholecystectomy

Section 1: Demographics	
1. Age	Under 18 | 18-24 | 25-34 | 35-44 | 45-54 | 55-64 | Above 65
2. Gender	Male | Female
3. Education level	High school or less | Diploma | Bachelor's degree | Master's degree | Doctorate or higher
4. Employment status	Employed full-time | Employed part-time | Unemployed | Student | Retired | Other
5. City of residence	Middle Province | Eastern Province | Northern Province | South Province | Western province | Others
6. Geographic location	Urban | Suburban | Rural

Section 2: General Questions	
How would you rate your overall satisfaction with the laparoscopic cholecystectomy procedure?	Very Satisfied | Satisfied | Neutral | Dissatisfied | Very Dissatisfied
Did you experience any complications or adverse effects?	Yes | No
How would you rate the improvement in your quality of life?	Significant Improvement | Moderate Improvement | Slight Improvement | No Change | Decline
Compared to your expectations, how would you rate the postoperative pain?	Much Less Than Expected | Less Than Expected | As Expected | More Than Expected | Much More Than Expected
Did you experience any limitations in daily activities?	Yes | No
How satisfied are you with the postoperative care?	Very Satisfied | Satisfied | Neutral | Dissatisfied | Very Dissatisfied
Any recurrence of gallstone-related symptoms?	Yes | No
How would you rate the financial burden?	Very Affordable | Affordable | Neutral | Expensive | Very Expensive
Any pre-existing conditions affecting recovery?	Yes | No
How well were you informed about the procedure's risks and benefits?	Very Well Informed | Well Informed | Moderately Informed | Poorly Informed | Not Informed at All

Section 3: Satisfaction Levels	
How would you rate communication with the surgical team?	Excellent | Good | Fair | Poor
Were you given adequate information regarding preoperative preparations?	Yes | No
How would you rate the responsiveness to your concerns before the procedure?	Very Responsive | Responsive | Neutral | Not Very Responsive | Not Responsive at All
Clarity of instructions for postoperative care?	Very Clear | Clear | Neutral | Unclear | Very Unclear
Did you feel supported during recovery?	Yes | No
How satisfied were you with the information about complications?	Very Satisfied | Satisfied | Neutral | Dissatisfied | Very Dissatisfied
How would you rate the healthcare team's professionalism and competence?	Excellent | Good | Fair | Poor
Was pain management addressed effectively?	Yes | No
Satisfaction with the nursing care?	Very Satisfied | Satisfied | Neutral | Dissatisfied | Very Dissatisfied
Would you recommend this procedure?	Definitely Yes | Probably Yes | Unsure | Probably No | Definitely No

Section 4: Quality of Life	
Rate your overall physical well-being:	Excellent | Good | Fair | Poor
Improvements in digestion and appetite?	Yes | No
Satisfaction with ability to engage in physical activities?	Very Satisfied | Satisfied | Neutral | Dissatisfied | Very Dissatisfied
Rate your mental and emotional well-being:	Excellent | Good | Fair | Poor
Any changes in sleep patterns or quality?	Yes | No
Satisfaction with energy levels and vitality?	Very Satisfied | Satisfied | Neutral | Dissatisfied | Very Dissatisfied
Did the procedure improve daily activities and tasks?	Yes, Significantly | Yes, Moderately | Yes, Slightly | No Change | Decline
Rate your social interactions and relationships:	Excellent | Good | Fair | Poor
Negative effects on body image or self-esteem?	Yes | No
Overall satisfaction with quality of life after the procedure?	Very Satisfied | Satisfied | Neutral | Dissatisfied | Very Dissatisfied

## References

[1] Mirghani HO, Aljuhani KF, Albuhairy AH (2022). Gallbladder stones and their contributing factors in Saudi Arabian population: knowledge and awareness assessment. Med Sci.

[2] Hassler KR, Collins JT, Philip K, Jones MW (2023). Laparoscopic cholecystectomy. StatPearls [Internet].

[3] Lombardo S, Rosenberg JS, Kim J (2018). Cost and outcomes of open versus laparoscopic cholecystectomy in Mongolia. J Surg Res.

[4] McIntyre Jr RC, Zoeter MA, Weil KC, Cohen MM (1992). A comparison of outcome and cost of open vs. laparoscopic cholecystectomy. J Laparoendosc Surg.

[5] Atif QA, Khan MA, Nadeem F, Ullah M (2022). Health-related quality of life after laparoscopic cholecystectomy. Cureus.

[6] Mattila K, Lahtela M, Hynynen M (2012). Health-related quality of life following ambulatory surgery procedures: assessment by RAND-36. BMC Anesthesiol.

[7] Carraro A, Mazloum DE, Bihl F (2011). Health-related quality of life outcomes after cholecystectomy. World J Gastroenterol.

[8] Lamberts MP, Özdemir C, Drenth JP, van Laarhoven CJ, Westert GP, Kievit W (2017). Cost-effectiveness of a new strategy to identify uncomplicated gallstone disease patients that will benefit from a cholecystectomy. Surg Endosc.

[9] Farahat T, Soltan HM, Shaheen HM, Hegazy NN, Elghalban HA (2021). Determinants of quality of life after laparoscopic cholecystectomy. Egypt J Hosp Med.

[10] Bagepally BS, Sajith Kumar S, Natarajan M, Sasidharan A (2022). Incremental net benefit of cholecystectomy compared with alternative treatments in people with gallstones or cholecystitis: a systematic review and meta-analysis of cost-utility studies. BMJ Open Gastroenterol.

